# What is your diagnosis?

**DOI:** 10.4274/jtgga.2018.0063

**Published:** 2018-08-06

**Authors:** Seyit Ahmet Erol, Cem Yaşar Sanhal, Yavuz Yılmaz, Dilek Şahin

**Affiliations:** 1 Clinic of Perinatology, University of Health Sciences, Zekai Tahir Burak Women’s Health Practice and Research Center, Ankara, Turkey; 2 Clinic of Pediatric Surgery, University of Health Sciences, Zekai Tahir Burak Women’s Health Practice and Research Center, Ankara, Turkey

A 25-year-old gravida 4, parity 1, abortion 2 patient was referred to our clinic during the 24^th^ gestational week due to abdominal echogenicity and intestinal cyst. An ultrasound examination revealed a cystic structure with minimal intestinal dilatation at the umbilical cord entry level of the fetal abdomen (Figure 1). Fetal growth was compatible with the gestational week, and amniotic fluid index and placental constitution were normal. Additional anomalies or fetal ascites were not detected on the detailed ultrasonography of the fetal anatomy. Antenatal chromosomal screening tests were normal. TORCH and parvovirus studies were negative. Cytogenetic analysis was suggested, but the patient did not accept the amniocentesis. Magnetic resonance imaging (MRI) T2-weighted images at 24 weeks of gestation showed a 30×29×22-mm cystic mass with internal septations and calcifications at the umbilical cord entry level of the lower fetal abdomen, which was evaluated as a meconium pseudocycst (Figure 2). The remaining abdominal organs and urinary system were normal. At the 28^th^ gestational week, we observed dilated bowel loops with the widest diameter of 20 mm, containing intraabdominal calcified areas on ultrasonographic examination (Figure 3). Meconium pseudocycst with meconium peritonitis secondary to jejunoileal atresia was considered. Pregnancy follow-ups were continued with weekly fetal well-being tests. Preoperative ultrasonographic examination showed a 370-mm fetal abdominal circumference and an enlarged intestinal ring of 30 mm in diameter with intraabdominal calcifications (Figure 4). Pregnancy was terminated by cesarean section at 38 gestational weeks after consulting with the pediatric surgery and neonatology departments. The newborn was delivered with meconium-stained amniotic fluid and the abdomen was distended. A few meconium passage was observed in the rectal examination. On the first day of labor, laparotomy was performed by pediatric surgery due to intestinal atresia. Intraoperative diffuse intestinal adhesions were detected. Jejunoileal atresia at 70 cm distal to the ligament of Trietz was accompanied with meconium peritonitis and bowel reanastomosis was performed (Figure 5). The neonate tolerated feeding very well and defecated. Current follow-ups continued successfully in the neonatal intensive care unit and the baby was discharged after one week. When the newborn was at two months of age, genetic analysis for cystic fibrosis (CF) was performed, and the result was negative.

## Answer

Other than meconium pseudocyst, possible diagnosis of abdominal cysts contains a wide range of diseases such as omental and mesenteric cysts, intestinal duplication cysts, obstructive uropathy, choledochal, pancreatic, urachal, renal and ovarian cysts, hydrometrocolpos, imperforate anus, Meckel diverticulum, ureterocele and sacrococygeal teratomas (1). Meconium ileus, volvulus, intestinal stenosis, atresia, intussusception and mesenteric vascular insufficiency could be a reason for intestinal perforation (2). Meconium pseudocyst occurs as a consequence of meconium extravasation secondary to intestinal perforation during the antenatal period, and it is observed as a hypoechoic mass attached to the extraluminal meconium (3,4). Meconium pseudocyst can rupture during pregnancy, albeit rarely (5). Meconium peritonitis is a chemical, sterile peritonitis induced by meconium spilling in the peritoneal cavity secondary to intrauterine intestinal perforation. Its prevalence is about 1 per 35,000 live births. Meconium peritonitis presents with common ultrasonographic signs as ascites, calcifications, polyhydramnios, meconium pseuodocysts, and bowel dilatation clinically (6). Meconium ileus occurs in about 10% to 15% of newborn with CF. Genetic analysis of CF is important and recommended (7). Magnetic resonance imaging (MRI) findings of meconium pseudocyst have been presented infrequently but it could be useful and valuable in the differentiation from other abdominal cystic masses (8,9,10). In the present case, both ultrasonographic and MRI scans revealed a hypoechoic mass with calcified echogenic organization in lower fetal abdomen. Progression of ileal atresia may occur with the rupture of meconium pseudocyst during pregnancy (5). In conclusion, meconium pseudocyst should be considered in the differential diagnosis when abdominal cystic mass is accompanied by intraabdominal calcifications. Therefore, prenatal diagnosis and follow-ups are important to diagnose likely intestinal atresia accompanied with meconium pseudocyst.

## Figures and Tables

**Figure 1:**
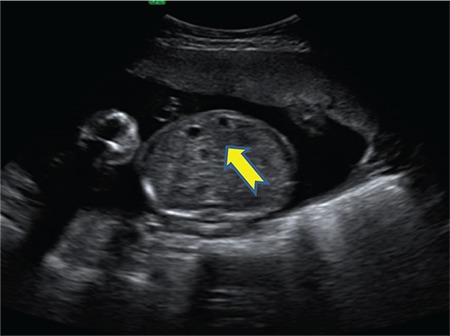
Ultrasound examination revealed a cystic structure with minimal intestinal dilatation at the umbilical cord entry level of the fetal abdomen

**Figure 2:**
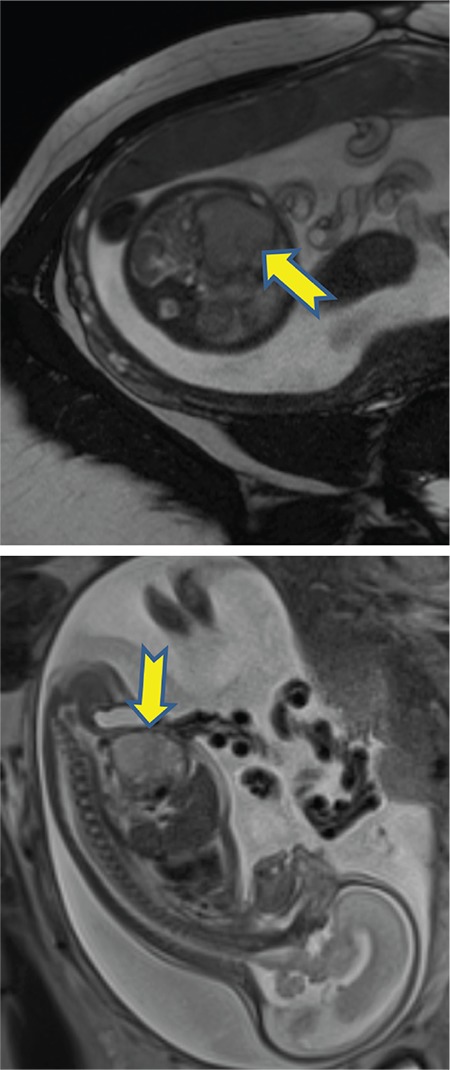
Magnetic resonance imaging T2-weighted images at 24 weeks of gestation showed a 30×29×22 mm cystic mass with internal septations and calcifications at umbilical cord entry level of fetal lower abdomen which was evaluated as meconium pseudocycst

**Figure 3:**
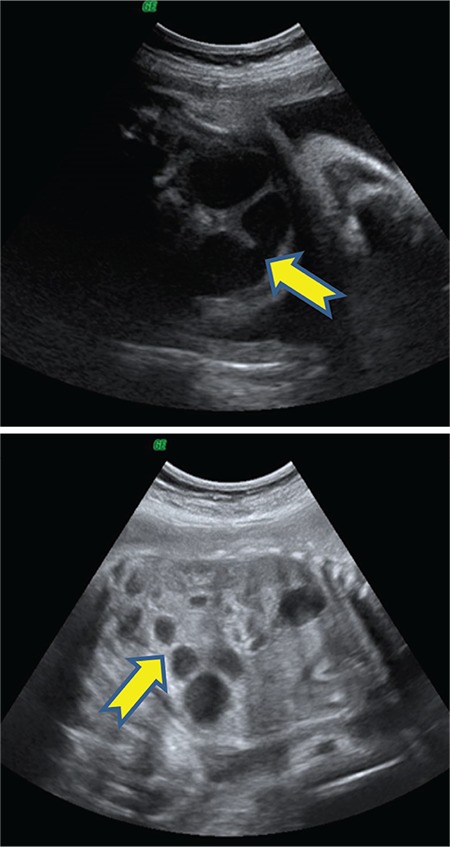
At the time of the 28^th^ gestational weeks, we observed dilated bowel loops with the widest diameter of 20 mm, containing intraabdominal calcified areas on ultrasonographic examination

**Figure 4:**
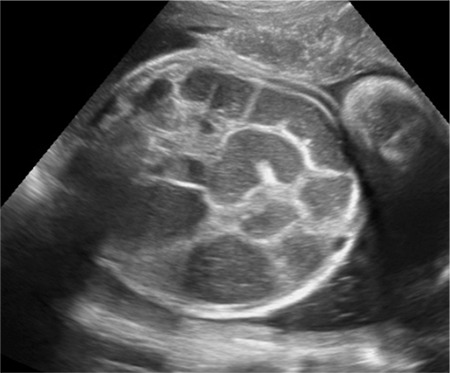
Preoperative ultrasonographic examination showed a 370 mm fetal abdominal circumference and an enlarged intestinal ring of 30 mm in diameter with intraabdominal calcifications at 38^th^ gestational weeks

**Figure 5:**
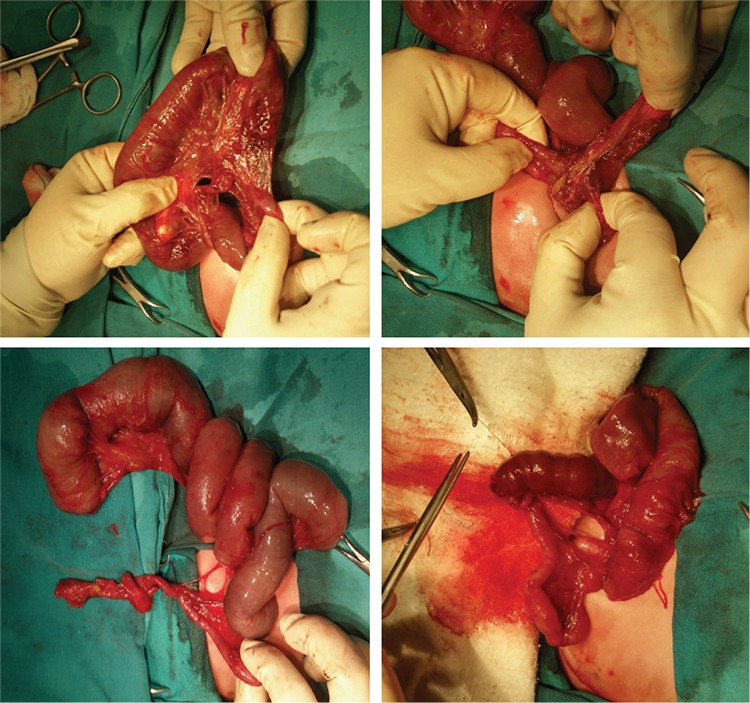
Jejunoileal atresia at 70 cm distal to the ligament of Trietz was located with meconium peritonitis and bowel reanastomosis was performed

## References

[1] Valladares E, Rodríguez D, Vela A, Cabre S, Lailla JM (2010). Meconium pseudocyst secondary to ileum volvulus perforation without peritoneal calcification: a case report. J Med Case Rep.

[2] Forouhar F (1982). Meconium peritonitis. Pathology, evolution, and diagnosis. Am J Clin Pathol.

[3] Nyberg DA, McGahan JP, Pretorius DH, Pilu G (2003). Chapter 13; Abdomen and gastrointestinal tract. Diagnostic imaging of fetal anomalies. Philadelphia: Lippincott Williams & Wilkins;.

[4] Eckoldt F, Heling KS, Woderich R, Kraft S, Bollmann R, Mau H (2003). Meconium peritonitis and pseudo-cyst formation: prenatal diagnosis and post-natal course. Prenat Diagn.

[5] Nakajima Y, Masaoka N, Asanuma A, Sone K, Nagaishi M, Miyakawa Y, et al (2011). A large meconium pseudocyst that developed into the generalized type during the antepartum period. J Med Ultrason (2001).

[6] Zangheri G, Andreani M, Ciriello E, Urban G, Incerti M, Vergani P (2007). Fetal intra-abdominal calcifications from meconium peritonitis: sonographic predictors of postnatal surgery. Prenat Diagn.

[7] Boue A, Muller F, Nezelof C, Oury JF, Duchatel F, Dumez Y, et al (1986). Prenatal diagnosis in 200 pregnancies with a 1-in-4 risk of cystic fibrosis. Hum Genet.

[8] Shinmoto H, Kuribayashi S (2003). MRI of fetal abdominal abnormalities. Abdom Imaging.

[9] Simonovsky V, Lisy J (2007). Meconium pseudocyst secondary to ileal atresia complicated by volvulus: antenatal MR demonstration. Pediatr Radiol.

[10] Wong AM, Toh CH, Lien R, Chao AS, Wong HF, Ng KK (2006). Prenatal MR imaging of a meconium pseudocyst extending to the right subphrenic space with right lung compression. Pediatr Radiol.

